# Eddy Current Sensor Array for Electromagnetic Sensing and Crack Reconstruction with High Lift-Off in Railway Tracks

**DOI:** 10.3390/s24134216

**Published:** 2024-06-28

**Authors:** Yuchun Shao, Zihan Xia, Yiqing Ding, Bob Crocker, Scott Saunders, Xue Bai, Anthony Peyton, Daniel Conniffe, Wuliang Yin

**Affiliations:** 1 School of Electrical and Electronic Engineering, University of Manchester, Manchester M13 9PL, UK; yuchun.shao@manchester.ac.uk (Y.S.); yiqing.ding@postgrad.manchester.ac.uk (Y.D.); xue.bai-10@postgrad.manchester.ac.uk (X.B.); a.peyton@manchester.ac.uk (A.P.); daniel.conniffe@manchester.ac.uk (D.C.); wuliang.yin@manchester.ac.uk (W.Y.); 2 Sperry Rail International Ltd., Derby DE24 8UP, UK; bobcrocker@btinternet.com (B.C.); ssaunders@sperryrail.com (S.S.)

**Keywords:** eddy current testing, electromagnetic induction, planar structure, theoretical calculation, measurement

## Abstract

A reliable and efficient rail track defect detection system is essential for maintaining rail track integrity and avoiding safety hazards and financial losses. Eddy current (EC) testing is a non-destructive technique that can be employed for this purpose. The trade-off between spatial resolution and lift-off should be carefully considered in practical applications to distinguish closely spaced cracks such as those caused by rolling contact fatigue (RCF). A multi-channel eddy current sensor array has been developed to detect defects on rails. Based on the sensor scanning data, defect reconstruction along the rails is achieved using an inverse algorithm that includes both direct and iterative approaches. In experimental evaluations, the EC system with the developed sensor is used to measure defects on a standard test piece of rail with a probe lift-off of 4–6 mm. The reconstruction results clearly reveal cracks at various depths and spacings on the test piece.

## 1. Introduction

Rail safety inspection has gained significant attention across multiple industries due to past incidents, driving the development and exploration of advanced detection methods and maintenance practices. Ratcheting, occurring within a spacing of 0.8 to 20 mm [1,2,3,4], may lead to the formation of closely spaced cracks. This process is often initiated by the continuous passage of train wheels over rail tracks, potentially resulting in critical safety hazards.

Eddy current (EC) testing is a widely used non-destructive testing (NDT) technique, distinguished by its capability of identifying surface and subsurface defects within rails without direct contact. This technique can be adapted for delivering instantaneous, real-time feedback and necessitates negligible surface preparation, streamlining the inspection process [1,5]. However, EC testing has limitations that affect the performance of rail inspection, including a reduction in signal and spatial resolution at high liftoff [6,7]. In response to the inherent limitations, many innovative solutions have been studied.

Li et al. developed an EC system with an increased coil gap to compensate for signal attenuation, allowing for accurate measurement of crack depth and inclination at the working frequency of 17 kHz [8]. However, a large coil gap can lead to a reduction in spatial resolution. Xu et al. proposed a differential EC system that can be applied to evaluate surface opening defects up to 8 mm deep with a maximum 15% relative error at a frequency of 60 kHz [9]. However, the spatial resolution is not quantified as the samples have a 100 mm crack spacing.

Kwon et al. proposed a 16-channel eddy current flaw detection system that works at 300 kHz with a lift-off of 1 mm and a high reliability of 1 mm margin of error [10]. However, maintaining a very low lift-off distance during rail inspection can increase the risk of probe damage or rail surface wear. Blitz et al. used differential-coil probes for detecting surface cracks in steel with a high lift-off of up to 12 mm, while there are limitations in accurately resolving closely spaced defects, as evidenced by 100 mm crack spacings [11].

To enhance the spatial resolution of the signal, Sukhanov et al. present a visualization technique for small heterogeneities in flat metallic objects using an eddy current sensor. It employs differential and flat spiral coils. The technique improves image resolution via post-processing. Spatial linear filtration and regularization are key to enhancing signal-to-noise ratios. Experimentally, for metallic objects at a 15 mm range, the method led to an increase in reconstructed signal resolution by up to 2.6 times relative to the raw measured data [12]. The sensor employed is limited to a single channel, necessitating the use of a scanner to comprehensively survey the desired area, which constrains the overall efficiency of the investigation.

Minimizing lift-off effects helps preserve spatial resolution. Meng et al. presented a simplified algorithm. It uses a multifrequency sensor to reduce lift-off impact in eddy current testing. This method tests nonmagnetic coatings on ferromagnetic substrates. It achieves up to 3% accuracy in thickness measurements for lift-offs up to 10 mm [13]. A significant limitation of using multi-frequency sensors for rail defect detection is their challenge in differentiating signals from various defect types due to the distinct interactions of each frequency with the substrate. This issue requires complex signal processing for accurate defect identification, increasing computational demands [14,15]. Additionally, the sensor’s performance at different frequencies can be affected by external factors, including temperature and humidity, complicating the defect detection process.

In this study, a multi-channel EC sensor array is produced to examine the cracks on rails. Previous studies explored the development of eddy-current sensors. The adaptive probe was proposed to change measurement resolution through changes in the transducer’s dimension by using multi-section excitation coils and the variable geometry of a core [16]. A matrix-type EC probe with a single transmitting coil, multiple receiving coils, and a ferrite core was implemented in the study [17]. These EC probes were designed to detect defects in certain dimensions. To distinguish the cracks on a railway with 5 mm of space, the dimensions of the coils and the space between them need to be much smaller, which was not achieved in previous studies. The EC system operates at 200 kHz with a 4–6 mm probe lift-off. The system can obtain data from any combination of the coil pairs. Therefore, the received data for coil combinations with multiple coil spacings and orientations can be evaluated. From measurements, the defects are reconstructed by solving an inverse problem with direct and iterative image reconstruction algorithms. Its capability to accurately identify and map cracks of various depths and intervals is demonstrated. This design effectively balances spatial resolution and lift-off distance, potentially making it suited for future railway inspection applications, and it can resolve cracks spaced as close as 5 mm.

## 2. Method

### 2.1. Measurement Setup for Rail Testing

**Figure 1:**
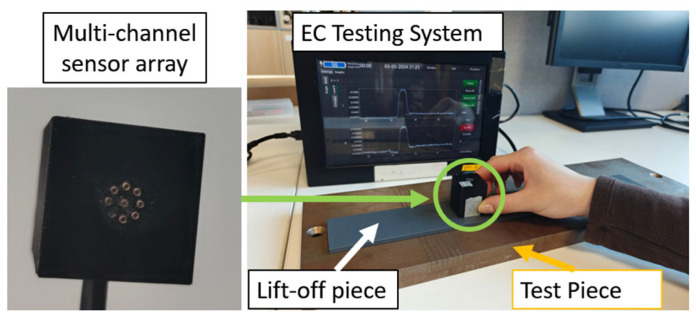
Schematic of rail crack scanning.

**Figure 2:**
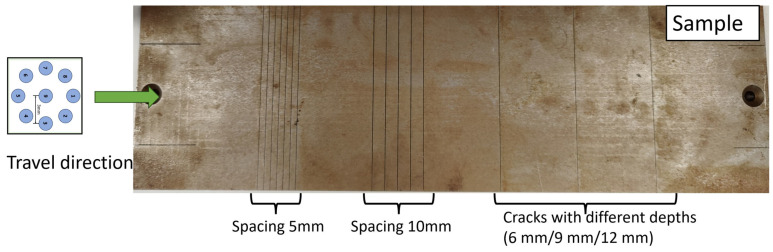
Illustration of line scanning along the sample.

Images of rail crack testing are shown in Figure 1. The customized multi-channel EC instrument, previously reported [18], is employed to acquire the EC probe signals from the coil array. The probe is moved above the testing sample across the cracks illustrated in Figure 2. This sensor can provide 36 independent measurements from all various coil combinations, featuring multiple coil spacings and sensitive orientations. By scanning once, abundant information about the defects can be collected for further analysis.

**Figure 3:**
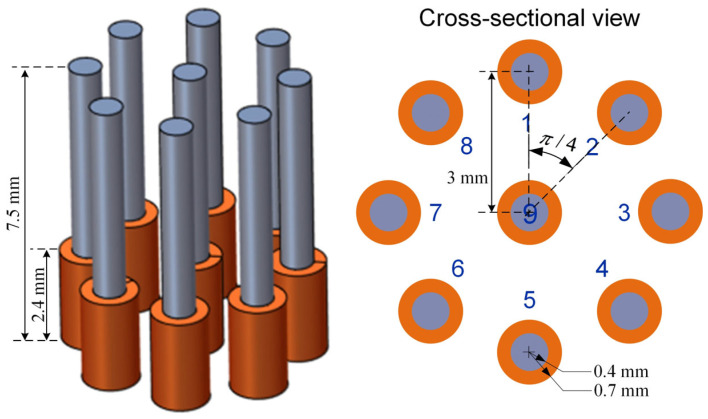
Basic structure of the EC probe.

The cracks on the rail could have various orientations, depths, and spaces between them. As illustrated in Figure 2, the observed cracks on the steel sample (grade R220). can be categorized into three distinct groups. The first group exhibits a spacing of 5 mm and a depth of 9 mm. In the second group, there are five cracks with a spacing of 10 mm and a depth of 9 mm. Furthermore, the third group comprises three cracks, each characterized by varying depths (6 mm, 9 mm, and 12 mm). To test the cracks with depth and spacing in the range of $d\in \left[6,\;9\right]$ mm and $s\in \left[5,\;20\right]$ mm, respectively, the ferrite-core coil array shown in Figure 3 was designed, which is symmetric and sensitive to cracks in various orientations. For testing a typical rail steel with an electrical conductivity of 4 MS/m and magnetic permeability of 40, an excitation frequency of 200 kHz, relating to the EC skin depth of 0.09 mm, is selected for rail surface crack detection. In principle, maintaining this frequency ensures fast data sampling rates even at high vehicle speeds, and at the same time, the frequency should not go further higher to avoid the reduction in sensor penetration depth caused by the skin effect phenomenon. For testing cracks on rail, the customized EC testing system can provide signals between all independent coil pairs at a speed of 127 frames per second. To ensure space between adjacent frames smaller than 5 mm, the speed should be lower than 0.635 m/s, i.e., 2.28 km/h. Of course, this is just an experimental setup. In a normal operation, 10 pairs of coil measurements are normally adopted, and not all combinations are necessary, which would give a speed of 36 km/h if the distance between adjacent frames of data is 5 mm. Similarly, it can be 72 km/h if the distance between adjacent frames of data is 10 mm.

Each coil consists of 200 turns of copper enameled wire with a diameter of 0.05 mm, as shown in Figure 1. In a rectangular coil cross-section, there are five layers, with 40 turns in each layer. The coil dimensions include an outer radius of 0.7 mm, an inner radius of 0.4 mm, and a height of 2.4 mm. A ferrite core is inserted in each coil to increase flux density. The implemented coil follows the previous study [18]. The amplitude of the excitation current is around 5 mA.

### 2.2. Forward Problem of Rail Testing

In the EC testing scenario, the governing diffusion equation of magnetic vector potential is as follows:

(1) $$\nabla^{2}A-j\omega \mu \sigma A=\mu_{0}J_{s}$$

where ω is the angular frequency, $J_{s}$ is the current density of the source field, μ and σ are magnetic permeability and electrical conductivity of the measurement space, respectively.

The electromagnetic field can be obtained by solving the diffusion equation, e.g., through the finite element method (FEM) and boundary element method (BEM), with the known source field excited by a coil array. For an array consisting of 9 coils, there are 36 independent measurements between coil pairs. The coil pairs have different spacing and orientations. The sensitivity of conductivity variation ∆σ on a plate due to defects, for certain coil pairs with index i,j, has been previously defined [19]:

(2) $$\lambda^{ij}=\int_{\Omega }S_{\sigma }^{ij}∆\sigma dV\;S_{\sigma }^{ij}=E^{i}\cdot E^{j}$$

where $\lambda^{ij}$ indicates the voltage variation on the *j*-th coil due to the excitation on the *i*-th coil, Ω is the region of the plate, and $E^{i}$ and $E^{j}$ are source fields excited by the *i*-th and *j*-th coils, respectively.

The integral in Equation (2) can be approximated by discretizing the plate domain into a variety of homogeneous voxels:

$$\lambda^{ij}\approx S_{\sigma }^{ij}∆\sigma$$

where $S_{\sigma }^{ij}\in C^{1\times n}$, $∆\sigma \in R^{n\times 1}$ is the conductivity distribution, *n* indicates the number of voxels.

Considering all independent measurements between k coils, with the number of measurements being m=k(k−1)/2, the matrix form holds for the following:

(3) $$\lambda \approx S_{\sigma }∆\sigma$$

where $\lambda =(\lambda^{ij})\in C^{m\times 1}$ is the measurement vector, $S_{\sigma }=(S_{\sigma }^{ij})\in C^{m\times n}$ is the sensitivity matrix. For simplicity, the sensitivity $S_{\sigma }$ and conductivity variation ∆σ are represented by S and σ hereinafter.

**Figure 4:**
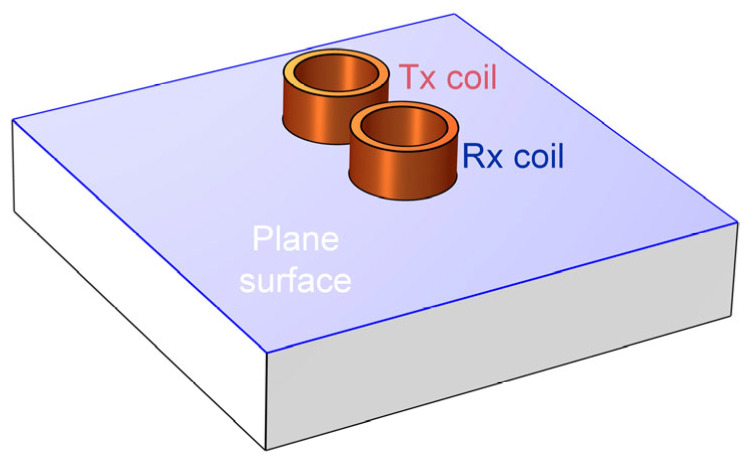
Surface of a conductive plane to evaluate the sensitivity between Tx and Rx coils.

**Figure 5:**
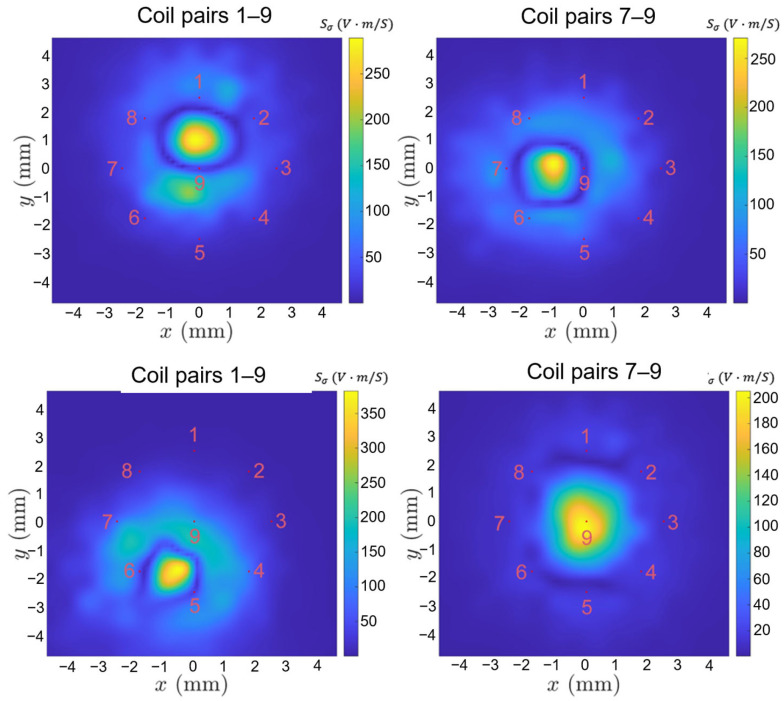
Sensitivity of conductivity for $S_{\sigma }^{19}$, $S_{\sigma }^{79}$, $S_{\sigma }^{56},$ and $S_{\sigma }^{15}$ (colour map indicates the value of the sensitivity with unit volt∙m/S, number is the index of coils). Red points indicate the positions of ferrite-core coils.

On the surface of the tested plane shown in Figure 4, the sensitivity of conductivity $S_{\sigma }^{ij}$ between representative coil pairs is shown in Figure 5. The electric field intensity in Equation (1) is calculated by the COMSOL 6.0 software, in which the electrical conductivity and relative magnetic permeability of the tested plate are 2 MS/m and 100, and the relative magnetic permeability of ferrite cores is 2000. There exists a high-value region of sensitivity between the transmitting (Tx) and receiving (Rx) coils. The sensitivity maps show that the measurements of different coil pairs contain complementary areas on the plane. By combining these measurements, the details of the cracks could be reconstructed.

In general, at lower lift-offs, the closely placed pairs work well, but as we increase lift-off, the far-placed pairs work well due to their larger sensitivity range. 

This study implemented all independent measurements between various coil pairs (36 measurements from the 9-coil probe) to reconstruct the cracks. Each coil pair has a distinct sensitive region, as shown in Figure 5, which contains the unique prior information for image reconstruction.

Without prior knowledge of the lift-off and crack, which is the case in real applications, it is best to select all combination coil pairs for crack reconstruction, as each of them may contribute to reconstruction.

### 2.3. Inverse Problem of Crack Reconstruction

Direct and iterative approaches based on sensitivity can be employed to reconstruct the conductivity variation, and reflect the distribution of defects on rail. Several representative direct methods, i.e., linear back projection (LBP), truncated singular value decomposition (TSVD), Tikhonov regularization, iterative algorithms, simultaneous iterative reconstruction technique (SIRT), Newton–Raphson (NR), and conjugate gradient (CG), are investigated for reconstruction of the relative change of conductivity.

The LBP algorithm reconstructs the conductivity distribution through a linear mapping from the measurement vector space to the conductivity distribution space:

$$g=S^{T}\lambda$$

where g indicates the reconstructed conductivity distribution.

The singular value decomposition provides a means to obtain the solution of conductivity distribution directly. If the rank of sensitivity matrix S is p, p=min⁡{m,n}, it can be decomposed into $S=U\Lambda V^{T}$, $U=\left[u_{1},u_{2},\ldots ,u_{m}\right]\in C^{m\times m}$**,** $V=\left[v_{1},v_{2},\ldots ,v_{n}\right]\in C^{n\times n}$ and $\Lambda =diag\left(\delta_{1},\delta_{2},\ldots ,\delta_{p}\right)\in R^{m\times n}$ with all components zero except for those diagonal components, diag(·) indicates the vector-to-matrix diag operator.

The TSVD algorithm employs

$$g=S^{+}\lambda ,S^{+}=V{\tilde{\Lambda }}^{-1}U^{T}$$

where ${\tilde{\Lambda }}^{-1}=\mathrm{diag}(w_{1}/\delta_{1},w_{2}/\delta_{2},...,w_{p}/\delta_{p})$, $w_{1},w_{2}$ are filtering parameters to reduce the influence of small singular values.

The Tikhonov method is one of the most commonly used universal regularization tools for solving ill-posed inverse problems. Based on the standard Tikhonov regularization procedure, the solution of equation λ+e=Sg, e indicates the measurement error vector, is expressed as follows:

$$g=(S^{T}S+\beta I{)}^{-1}S^{T}\lambda$$

where I is the identity matrix and β is the regularization parameter. More details on direct methods can be found in [20,21].

The iterative approaches optimize the reconstructed conductivity in an iterative manner:

(4) $$g_{k+1}=g_{k}+\Delta g_{k}$$

where k is the iterative step.

The NR algorithm can find the root of the non-linear function for the least squares problem ${\left\|Sg-\lambda \right\|}_{2}^{2}$, by approaching the local minimum iteratively. An appropriate initial guess is required to reach the global minimum by the NR algorithm. SIRT is commonly used for image reconstruction in X-ray computerized tomography and was introduced to electromagnetic tomography. The algorithm usually requires a large number of iterations, and its convergence rate is relatively low. The updating directions of the NR and SIRT algorithms are shown in Table 1.

The CG algorithm updates the variable following the orthogonality of residuals and the conjugacy of the search directions. The algorithm is characterized by its fast convergence rate while suffering from measurement noise. Setting the initial conditions $g_{0}=S^{T}\lambda ,$ $r_{0}=S^{T}\lambda -Ag_{0}$ and $p_{0}=r_{0}$, the implementation of the CG algorithm for conductivity reconstruction is expressed by the following:

$$\Delta g_{k}=\alpha_{k}p_{k}\;\alpha_{k}=\frac{{r_{k}}^{T}r_{k}}{{p_{k}}^{T}Ap_{k}}\;r_{k+1}=r_{k}-\alpha_{k}Ap_{k}\;\beta_{k}=\frac{{r_{k+1}}^{T}r_{k+1}}{{r_{k}}^{T}r_{k}}\;p_{k+1}=r_{k+1}+\beta_{k}p_{k}$$

The optimization repeats until the iterative step reaches its maximum value or the residues fall below the threshold. Comprehensive analysis of the CG, NR, and SIRT reconstruction algorithms can be found in the previous studies [22,23], which is briefly described here to avoid redundancy.

To obtain the 1-D scanning results of defect distribution, the arithmetic mean of 2-D reconstruction results is aligned into a vector with elements corresponding to each testing point of the probe, $v=v_{i}\in R^{l\times 1}$, l represents the number of testing points.

**Figure 6:**
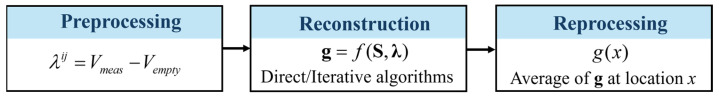
The flowchart of data processing.

The flowchart of data processing is shown in Figure 6 below.

The employed field-quantity method for the forward problem and direct and iterative algorithms for the inverse problem are presented in a concise manner.

The methods we proposed are effective. Furthermore, the realization of crack reconstruction in combination with the miniature probe and customized EC system is one of the main highlights of the paper. We make it possible to obtain the signals that clearly distinguish the closely spaced cracks and variations in depth for the first time.

## 3. Results and Discussion

All potential transmitter and receiver coil pairings (shown in Appendix A) were thoroughly evaluated, with data collected throughout the experiment. The influences of probe lift-off, spatial resolution of defects, and the effectiveness of the implemented inverse algorithm for crack reconstruction are discussed in detail.

### 3.1. Received Data

**Figure 7:**
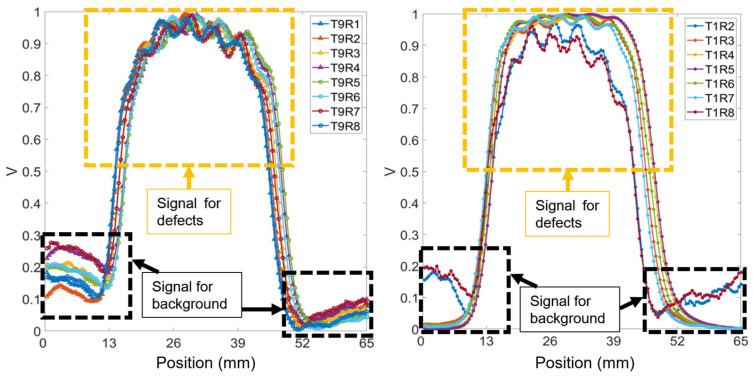
Normalized received signals of 15 T–R pairings for seven cracks at 5 mm spacing, at 5 mm lift-off, and 200 kHz.

Theoretically, if adjacent cracks on a rail are closely spaced, the response signals from these cracks can merge together, resulting in a single signal that displays as a continuous high value range due to low spatial resolution. Figure 7 presents the normalized voltage signals obtained by scanning across seven cracks spaced 5 mm apart, with a 5 mm lift-off, at a frequency of 200 kHz. The seven peaks of the response signal are represented as the small peaks on the bump. This figure presents data from 15 of the possible 36 transmitter (T) and receiver (R) coil combinations contained in the multichannel EC array. Each plot within the figure shows the response signal for seven cracks, appearing as distinct peaks atop the main bump. The combinations from T9–R1 to T9–R8 have a consistent coil spacing of 3 mm. The pairings T1–R2 and T1–R8 feature the narrowest coil spacing at 2.296 mm, while T1–R5 has the widest spacing at 6 mm, as referenced in Figure 3. At a 5 mm lift-off distance, all coil spacings can detect a group of cracks. However, identifying the exact number of cracks requires further analysis through algorithms due to the fewer distinct peaks on the bump.

**Figure A1:**
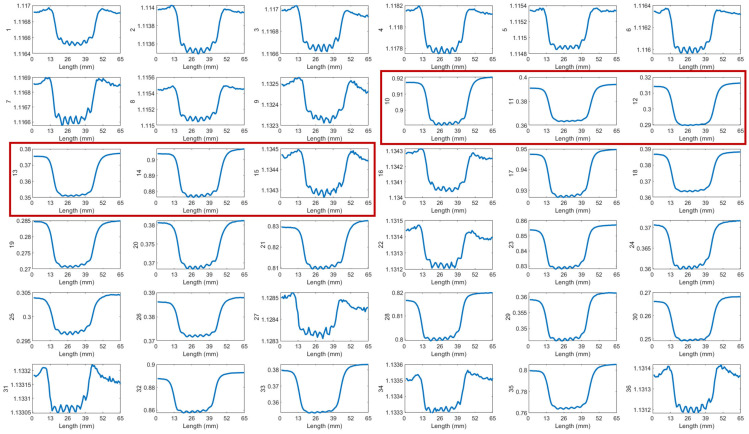
Received signals of 36 T–R pairings for seven cracks at 5 mm spacing, 4 mm lift-off, and 200 kHz. The plots in the red box are utilized for further data processing, with the results displayed in Figure 9.

**Figure A2:**
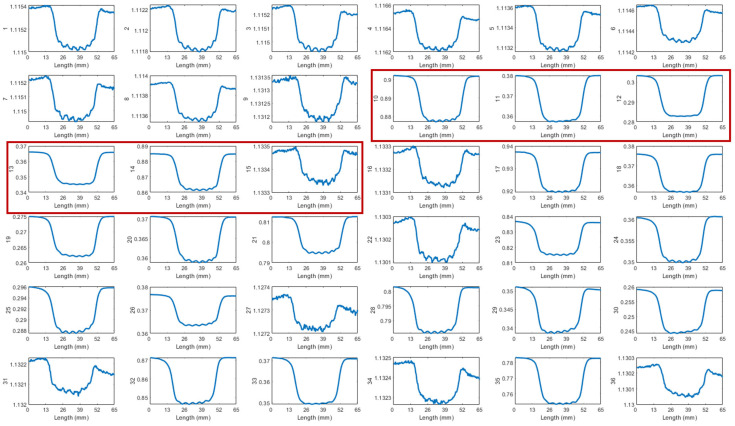
Received signals of 36 T–R pairings for 5 cracks at 5 mm spacing, 5 mm lift-off. and 200kHz. The plots in the red box are utilized for further data processing, with the results displayed in Figure 9.

**Figure A3:**
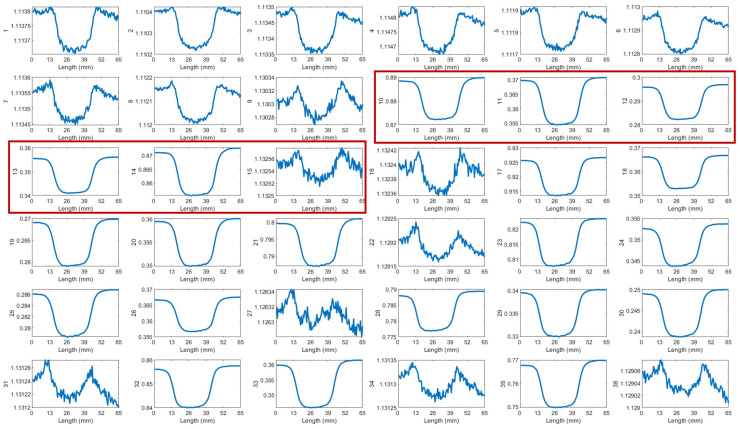
Received signals of 36 T–R pairings for seven cracks at 5 mm spacing, 6 mm lift-off. and 200 kHz. The plots in the red box are utilized for further data processing, with the results displayed in Figure 9.

Reducing the probe lift-off improves its spatial resolution. This study examines the balance between spatial resolution and lift-off effects by scanning a sample featuring cracks spaced 5 mm apart, with lift-offs varying between 4 mm and 6 mm. The raw data for all transmitter–receiver (T–R) pairings at 4 mm, 5 mm, and 6 mm lift-offs are illustrated in Figure A1, Figure A2 and Figure A3 in the Appendix A. At a 4 mm lift-off, the peaks corresponding to individual cracks are more obvious and can be observed visually. Signal amplitude is greater than at lift-offs of 5 mm and 6 mm. At a 6 mm lift-off, the signal for T–R pairings with smaller spacings becomes noisy, obscuring the peaks on the bump and reducing the amount of inspectable information.

The sensitive region varies for coil pairs in different spacing and locations, which mainly concentrates near the linking region between T–R pairs as shown in Figure 5. From the measurement signals in Figure A3, it is clear that the coil pairs with relatively small space are more sensitive to the closely spaced cracks, while those pairs with large spacing are less sensitive to distinguishing the closely spaced cracks but have a far wider range for deeper and individual cracks.

**Figure A4:**
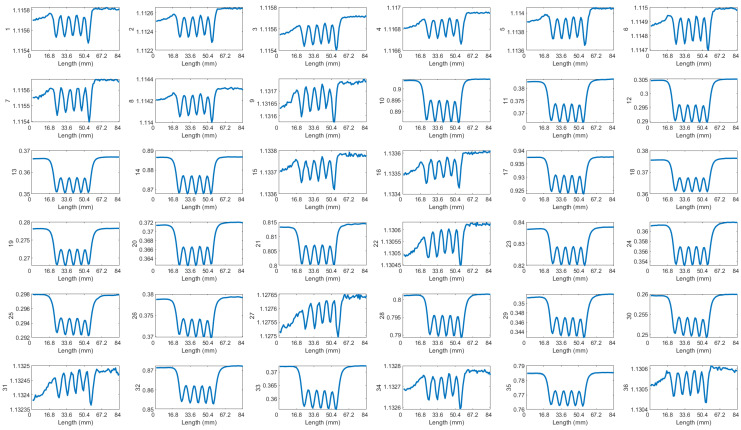
Received signals of 36 T–R pairings for 5 cracks at 10 mm spacing, 5 mm lift-off. and 200kHz.

**Figure A5:**
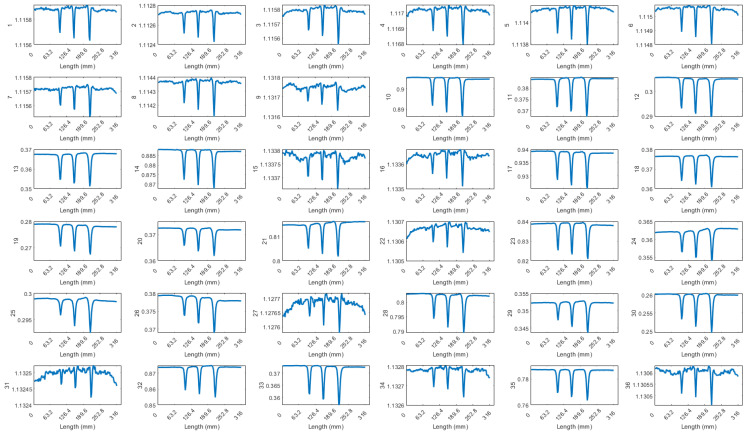
Received signals of 36 T–R pairings for three cracks with depths of 6, 9, 12 mm, 5 mm lift-off, and 200 kHz.

The data obtained from scanning five adjacent cracks, each separated by a 10 mm spacing, and three cracks with varying depths (6 mm, 9 mm, and 12 mm), with a 5 mm lift-off, are presented in Figure A4 and Figure A5 in the Appendix A. These results are encouraging for direct crack detection, as the cracks can be distinctly identified through five distinct peaks in the raw data. Furthermore, the capability to discern cracks of different depths is demonstrated by the amplitude variation of the response signal, where a shallower crack corresponds to a lower signal amplitude. This correlation between crack depth and signal amplitude indicates the effectiveness of the detection method in distinguishing cracks of varying severities directly from the raw data.

Distinguishing the response signals corresponding to specific cracks from the peak of the bump becomes challenging when the lift-off is set to 6 mm. In addition, there is a noticeable decrease in the amplitude of the crack signal peaks compared with 4 mm and 5 mm lift-off. The measurement signals at the 6 mm lift-off can detect the region of cracks, while the individual cracks can hardly be distinguished at this lift-off.

The signal-to-noise ratio (SNR) of different coil pairs is mainly influenced by factors including coil spacing (determining the amplitude of signals), symmetry of ferrite cores, fabrication of the handmade miniature probe, and consistency among various measurement channels in the customized EC system.

Although a smaller liftoff provides superior spatial resolution and signal strength, it poses a higher risk of damage to the delicate sensor probe, potentially increasing maintenance costs. Conversely, a larger liftoff introduces noise into the signal and captures less information. Consequently, a 5 mm lift-off is selected to balance spatial resolution and signal strength, mitigating the risk of probe damage and maintaining data quality.

### 3.2. Evaluation of Results Using Inverse Problems

There are differences in the results from different inverse algorithms, although they all try to find the solution method of the least-squares problem. Image reconstruction in electrical tomography, for example, has a similar pursuit where different algorithms have been developed over the years [20,21]. The inverse problem is non-linear, underdetermined, ill-conditioned, and ill-posed, which makes it difficult to obtain “the solution”. It is not a case of finding a simple solution in the least-squares sense.

The performance of various algorithms is different regarding the reconstructed crack depth, space, and lift-off. Adjusting parameters in the algorithm is another way to improve the reconstruction accuracy. The main point of this study is to reconstruct the distribution of cracks on rail samples by utilizing multiple combinations of measurements of the coil array, and the reconstruction method is implemented to improve the spatial resolution of crack reconstruction.

In this section, results using different inverse problem methods are evaluated and compared.

#### 3.2.1. Seven Cracks with 5 mm Spacing

**Figure 8:**
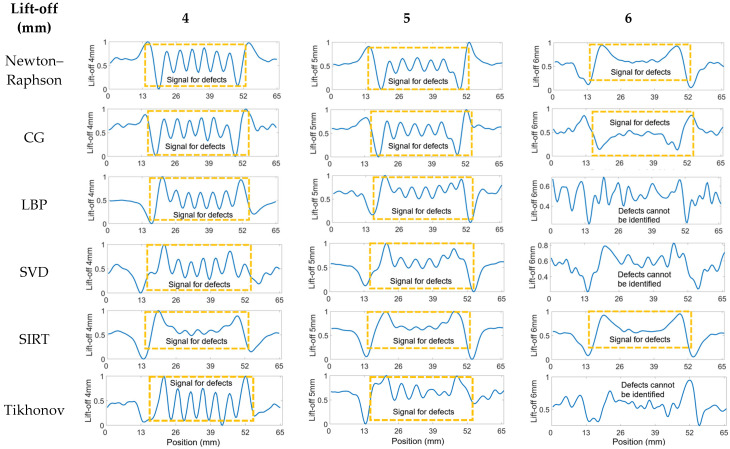
Processed signals of 36 T–R pairings for seven cracks at 4, 5, and 6 mm spacing and 5 mm lift-off.

The processed data utilizing all the algorithms can be observed in Figure 8. All 36 pairings of received signals are employed when applying inverse method algorithms. It can be noted that the signals for seven cracks, each spaced 5 mm apart, are distinguished from a significant bump into seven peaks when the lift-off is set to 4 mm with inverse algorithms applied. At a 5 mm lift-off, the Newton–Raphson, CG, and LBP methods continue to exhibit good results. However, when the lift-off increases to 6 mm, the crack signals are obscured by noise, rendering the plots devoid of useful information. The criterion for cracking is the visibility of clear individual peaks with clear intervals above the noise signals. Therefore, the preferred algorithmic methods are Newton–Raphson, CG, and LBP, with the processed signals at a lift-off of 4 mm, 5 mm, and 6 mm.

**Figure 9:**
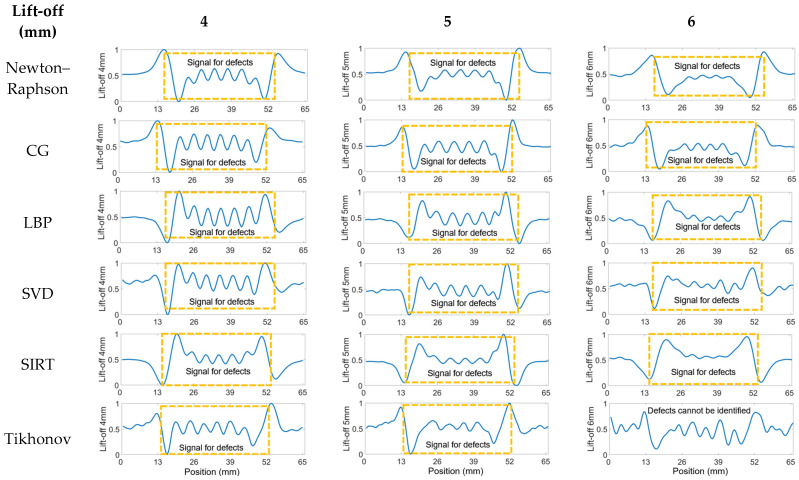
Processed signals of 10–14 T–R pairings for seven cracks at 4, 5, and 6 mm spacing and 5 mm lift-off.

Distinguishing the response signals corresponding to specific cracks from the peak of the bump becomes challenging when the lift-off is set to 6 mm. This difficulty is evident in Figure A3 in the Appendix A, where the received signals from coil pairs with shorter spacings are processed. The underlying issue is that the reduced distance between coils results in a diminished penetration depth, adversely affecting signal clarity and the ability to identify cracks accurately. Accordingly, pairings 10 to 14, characterized by their wider spacings, were selected for analysis across all algorithms. The outcomes of this evaluation are depicted in Figure 9, presented below. The analysis indicates a reduction in noise interference, allowing for the detection of cracks in the plots. Yet, determining the exact number of cracks remains challenging. Additionally, there is a noticeable decrease in the amplitude of the crack signal peaks at 4 mm and 5 mm lift-off, likely attributable to the diminished quantity of input signals available for data processing.

#### 3.2.2. Five Cracks with 10 mm Spacing

**Figure 10:**
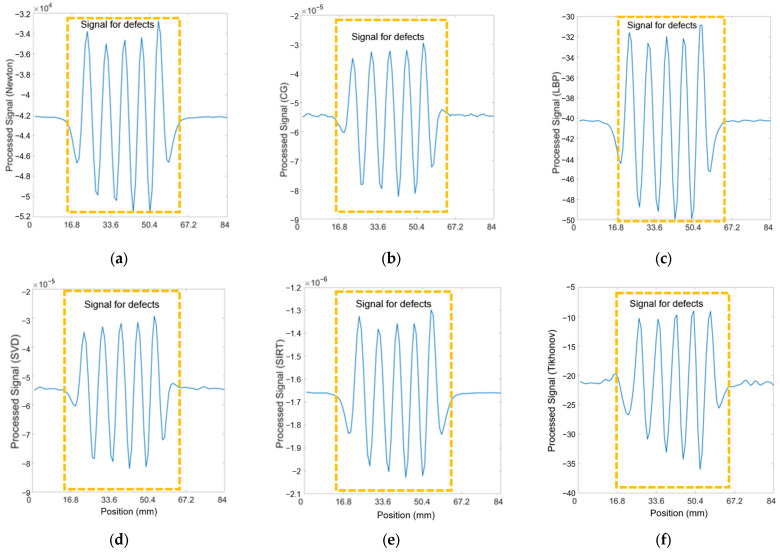
Processed signals of 36 T–R pairings for 5 cracks at 10 mm spacing, 5 mm lift-off, and 200 kHz using (**a**) Newton–Raphson (**b**) CG (**c**) LBP (**d**) SVD (**e**) SIRT (**f**) Tikhonov methods.

Given that the response signal can clearly identify cracks with a 10 mm spacing, it is crucial to assess whether the processing algorithms might alter the raw data. Figure 10 illustrates this point effectively, showing that all five cracks can be distinguished by their respective peaks in the processed data. This indicates the algorithms preserve the integrity of the raw signal, allowing for accurate identification of cracks, a vital aspect of the reliability of the detection method.

#### 3.2.3. Three Cracks with 6 mm, 9 mm, and 12 mm Depths

**Figure 11:**
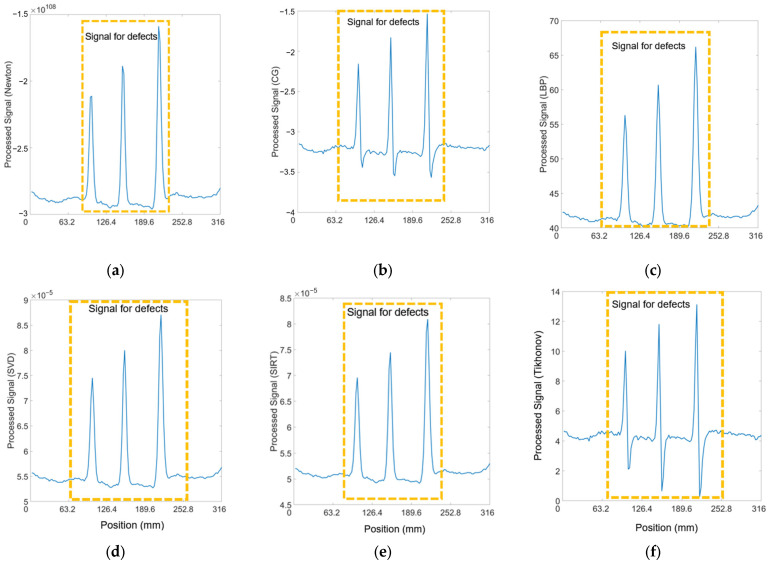
Processed signals of 36 T–R pairings for three cracks with 6, 9, and 12 mm depths at 5 mm lift-off and 200 kHz using (**a**) Newton–Raphson (**b**) CG (**c**) LBP (**d**) SVD (**e**) SIRT (**f**) Tikhonov methods.

The algorithms were also applied to signals received from three cracks of varying depths, aiming to determine if data processing impacts the signal’s sensitivity to crack depth. Figure 11 demonstrates that the peak amplitude of the signal for each crack correlates nearly directly with the crack’s depth. This correlation suggests that the algorithms effectively enable the assessment of cracks of different depths on railway tracks, providing a reliable means to gauge the severity of such defects based on signal amplitude analysis.

## 4. Conclusions

In conclusion, this research introduces an innovative approach to railway track crack detection, incorporating a nine-coil sensor array, a multi-channel instrument, and sophisticated inverse algorithms, specifically the Newton–Raphson, CG, and LBP algorithms. Operating at a frequency of 200 kHz and with a lift-off of 5 mm, this system achieves an optimal balance between lift-off and spatial resolution. It significantly enhances the detection of closely spaced cracks (as narrow as 5 mm apart) through refined data processing techniques, facilitating more accurate crack identification. The carefully chosen probe lift-off not only minimizes the risk of damage to the sensitive sensor probe but also ensures data quality. This approach does not compromise the system’s ability to detail intricate information about the defects. The processed data demonstrates the system’s success in distinguishing between cracks of various depths and intervals, highlighting the algorithms’ capacity to extract useful information from the raw signal. These findings show promise for realizing non-contact railway defect detection capabilities.

Future research will expand upon this work by exploring the integration of this system into railway test vehicles, assessing its performance in practical rail conditions, and further refining the sensing system and algorithms for increased performance.

## Figures and Tables

**Table 1:** Updating directions of the NR and SIRT algorithms for conductivity reconstruction.

**Algorithm**	$\mathrm{Updating}\;\mathrm{direction},\;{\Delta g}_{k}=d_{k}\left({Sg}_{k}-\lambda \right)$ $,\;d_{k}\in C^{n\times m}$
**NR**	$d_{k}=-{\left(S^{T}S\right)}^{-1}S^{T}$
**SIRT**	$d_{k}=-\gamma S^{T}{diag(diag*(SS^{T})}^{-1})(Sg_{k}-\lambda ),\gamma =10^{-3}/\max\{diag*(SS^{T})\}$, diag*(·) indicates the matrix-to-vector diag operator

## Data Availability

The datasets presented in this article are not readily available because the data are part of an ongoing study. Requests to access the datasets should be directed to the corresponding author, Zihan Xia (zihan.xia@manchester.ac.uk).

## Appendix A

This appendix presents a comprehensive illustration of the raw data collected by the sensing system, which is employed for data processing.
